# Hans Chinese consume less O_2_ for muscular work than european-american

**DOI:** 10.1186/s40779-024-00578-9

**Published:** 2024-11-21

**Authors:** Mei-Han Guo, David Montero

**Affiliations:** 1 Beth Israel Deaconess Medical Center, Harvard Medical School, Boston, MA 02115 USA; 2 Faculty of Medicine, Hong Kong University, Hong Kong, China; 3 Department of Medicine, School of Clinical Medicine, Hong Kong University, Hong Kong, China; 4 Libin Cardiovascular Institute of Alberta, University of Calgary, Calgary, T2N 4N1 Canada

**Keywords:** Hans Chinese, O_2_ uptake, Metabolic cost of exercise, Lean body mass, Body fat, European–American

Dear Editor,

The Hans Chinese (HC) ethnic group, comprising approximately 1.4 billion individuals, represents the largest workforce globally. Historically, HC has been predominantly isolated from other ethnic groups for over 3 millennia, resulting in distinct genetic and physiological characteristics [1, 2]. Consequently, the energy required to maintain essential functions, represented by the basal metabolic rate (BMR), cannot be accurately assessed in HC with algorithms developed for European-American (EA) populations, even when controlling for differences in body size [3]. Notably, the widely used Harris-Benedict equation tends to overestimate BMR, relative to measured BMR via indirect calorimetry, in more than 50% of HC individuals [3]. Hence, HC generally expends less energy than EA under basal (resting) conditions. This raises an important question: does HC require lower energy expenditure than EA for equivalent external work? The present aimed to address this inquiry, while adjusting for key confounding factors such as age, sex, physical activity, aerobic capacity, BMR, and body composition.

Healthy adult individuals (*n* = 194) from both EA and HC, spanning the adult life of 20 − 89 years, were recruited in Calgary (Canada) and Hong Kong (China) using equivalent online and printed advertisements in English and Traditional Chinese, respectively. The EA participants exhibited diverse European (Caucasian) ancestries due to Calgary’s demographic composition of primarily first- or second-generation immigrants. In contrast, most HC individuals were descendants of Cantonese immigrants, reflecting the lifestyle and dietary habits of the Guangdong province in China. According to the study design, EA and HC groups were matched for sex (*P* = 0.972), age (*P* = 0.282), and physical activity, as detailed in Table 1. Consequently, both groups demonstrated comparable aerobic training status as represented by peak O_2_ uptake relative to lean body mass [VO_2peak_/LBM)] (*P* = 0.737). All participants were required to self-identify their ethnicity as either EA or HC supported by legal documentation (Canadian/Hong Kong identity card), name, country of birth and residential history, along with confirmation that no known ancestor belonged to distinct ethnicities. This self-identification method has shown a very high percentage of agreement (> 90%) with genetic-based measures of ancestry specific to EA and HC populations [4]. Inclusion criteria comprised healthy status based on resting electrocardiogram (ECG)/echocardiography screening alongside health/clinical questionnaires. Exclusion criteria comprised presence of medical symptoms/medication and/or history of chronic disease. The study received approval from the Institutional Review Board at the University of Hong Kong/Hospital Authority West Cluster (UW 21–401).

**Table 1:** General characteristics, body composition and exercise economy in European–American (EA) and Hans Chinese (HC) individuals

Characteristic	EA (*n* = 94)	HC (*n* = 100)	*P*-value
Age (year, mean ± SD)	54.0 ± 16.7	51.5 ± 14.9	0.282
Sex [female, *n* (%)]	43 (45.7)	46 (46.0)	0.972
Body height (cm, mean ± SD)	172.2 ± 9.7	165.9 ± 8.5	< 0.001
Body mass (kg, mean ± SD)	71.3 ± 13.4	61.5 ± 9.9	< 0.001
BMI (kg/m^2^, mean ± SD)	23.9 ± 3.1	22.3 ± 2.8	< 0.001
BSA (m^2^, mean ± SD)	1.84 ± 0.21	1.68 ± 0.16	< 0.001
Hb (g/dl, mean ± SD)	14.2 ± 1.2	13.6 ± 1.6	0.006
SBP (mmHg, mean ± SD)	131.8 ± 21.0	120.4 ± 13.8	< 0.001
DBP (mmHg, mean ± SD)	77.3 ± 15.2	79.0 ± 10.2	0.357
Smoking (%)	0	0	1.000
BMR (kcal/d, mean ± SD)	1592 ± 245	1385 ± 198	< 0.001
MVPA (h/week, mean ± SD)	6.4 ± 3.4	6.0 ± 4.2	0.470
VO_2peak_ [ml/(min·kg), mean ± SD]	37.7 ± 11.1	36.1 ± 11.8	0.327
VO_2peak_/LBM [ml/(min·kg), mean ± SD]	48.2 ± 11.0	48.8 ± 12.7	0.737
Body composition			
BMC (kg, mean ± SD)	2.4 ± 0.5	2.1 ± 0.5	< 0.001
Body fat (kg, mean ± SD)	15.7 ± 5.1	15.5 ± 4.5	0.765
Body fat percentage (%)	22.4 ± 6.4	24.9 ± 6.8	0.014
LBM (kg, mean ± SD)	52.7 ± 11.1	45.0 ± 8.9	< 0.001
Exercise economy			
VO_2 100 W_ (ml/min, mean ± SD)			
Unadjusted	1352 ± 340	1227 ± 237	0.004
Adjusted by sex	1352 ± 290	1228 ± 290	0.003
Adjusted by MVPA	1352 ± 293	1227 ± 292	0.003
Adjusted by VO_2peak_	1350 ± 291	1230 ± 290	0.005
Adjusted by VO_2peak_/LBM	1350 ± 305	1219 ± 305	0.007
Adjusted by body mass	1332 ± 301	1246 ± 300	0.060
Adjusted by BMC	1342 ± 313	1225 ± 312	0.020
Adjusted by body fat mass	1347 ± 301	1228 ± 298	0.012
Adjusted by body fat percentage	1343 ± 310	1231 ± 298	0.020
Adjusted by LBM	1328 ± 310	1242 ± 308	0.085
Adjusted by BMR	1329 ± 304	1249 ± 303	0.084

*BMC* bone mineral content, *BMR* basal metabolic rate, *BMI* body mass index, *BSA* body surface area, *DBP* diastolic blood pressure, *Hb* hemoglobin, *LBM* lean body mass, *MVPA* moderate-to-vigorous physical activity, *SBP* systolic blood pressure, *VO*_*2100W*_ O_2_ consumption during cycling at 100 W, *VO*_*2peak*_ peak O_2_ consumption

Participants were instructed to refrain from engaging in strenuous exercise, as well as consuming alcohol and caffeine, for 24 h before testing. The timing of testing sessions was standardized between EA and HC groups. All measurements were performed after a fasting period of at least 4 h to mitigate postprandial hemodynamic alterations. BMR was evaluated using ethnicity-specific algorithms [5, 6]. An incremental exercise test was conducted on a supine leg cycle ergometer (KICKR Core, Wahoo, USA) to eliminate weight-bearing effects on exercise economy. The workload was progressively increased by 10−30 watts (W) increment every 50 s up to 100 W, which was kept constant until steady-state pulmonary O_2_ uptake (VO_2_) was achieved. Subsequently, the workload continued to increase in increments of 10 to 30 W every 50 s until exhaustion occurred. VO_2_, CO_2_ output, and ventilation were recorded throughout the exercise test by a mixing chamber system (KORR Medical, USA). Calibration of the gas analyzers and flowmeter was conducted prior to each test. VO_2_ and the respiratory exchange ratio were averaged over the final 15 s of each stage in accordance with current recommendations. Body composition was assessed via dual-energy X-ray absorptiometry (Hologic QDR 4500, Hologic, Hong Kong) [7]. Statistical analyses were performed using SPSS version 26.0 (IBM, USA). The independent *t*-test was employed for comparisons between EA and HC groups. A two-tailed *P*-value less than 0.05 indicated statistically significant.

The general characteristics of EA and HC participants are presented in Table 1. All individuals were non-smokers and classified as non-obese. Measures related to body size, along with BMR, were lower in HC compared with EA (*P* < 0.001). Regarding body composition, HC exhibited reduced bone mineral content (BMC, *P* < 0.001) and LBM (*P* < 0.001), as well as a higher percentage of body fat than EA (*P* = 0.014). Exercise economy, as reflected by VO_2_ at a workload of 100 W (VO_2 100 W_), differed between the two ethnic groups, with HC displaying lower VO_2 100 W_ than EA (*P* = 0.004), resulting in a gap of approximately 10% (Table 1). Adjustments for sex (*P* = 0.003), physical activity (*P* = 0.003), VO_2peak_ (*P* = 0.005), BMC (*P* = 0.020), or body fat mass (*P* = 0.012) did not alter the ethnic difference in VO_2 100 W_. Conversely, adjustment for BMR (*P* = 0.084) or LBM (*P* = 0.085) rendered the difference in VO_2 100 W_ between EA and HC statistically insignificant.

This study demonstrates that HC individuals consume less O_2_ for a given power output compared with EA counterparts matched by sex, age, physical activity level, aerobic capacity, and training status. Therefore, HC possesses a superior exercise economy, which may translate into lower energy costs during physically related military operations [8]. The observed ethnic disparity remains unchanged when adjusting for intrinsic sex differences in body fat mass or BMC, the latter of which has garnered increasing attention in the HC population [9, 10]. However, it is not fat mass nor BMC that accounts for this difference, but LBM, the component of body composition hypothesized to contribute to ethnic variations in O_2_ consumption. Exercise economy does not differ between EA and HC when adjusted for LBM. Another covariate known to vary between these groups is BMR, which also statistically nullifies the ethnic difference in exercise economy. Thus, the lower resting metabolism observed in HC could contribute to their reduced O_2_ consumption during exercise [3]. Nonetheless, this effect is likely to be minor, as the ethnic difference in BMR, when converted to O_2_ consumption, accounts for only approximately 18% of the overall gap in the exercise economy. Furthermore, the low *P-*values associated with the ethnic gap in exercise economy persist even after adjustments for LBM or BMR (Table 1), indicating that neither body composition nor BMR explains the majority of the observed ethnic dimorphism among comprehensively matched EA and HC individuals. It should be noted that relative to EA participants, HC exhibits more divergent genetic factors influencing exercise capacity than other major ethnic groups [2]. Genetic divergences between EA and HC include single-nucleotide polymorphisms related to metabolic pathways and energy expenditure, which could independently contribute to differences in exercise economy regardless of aerobic capacity [2]. Additionally, this study predominantly included middle-aged individuals who have been exposed longer than younger cohorts to environmental and epigenetic influences. Future research will mechanistically investigate the underlying causes contributing to the enhanced exercise economy observed in HC compared with EA.

## Data Availability

All data associated with this study will be available upon reasonable request to the corresponding author.

## Abbreviations

BMR: Basal metabolic rate
EA: European-American
HC: Hans Chinese
LBM: Lean body mass
